# Diurnal decline in photosynthesis and stomatal conductance in several tropical species

**DOI:** 10.3389/fpls.2023.1273802

**Published:** 2023-10-24

**Authors:** Wachira Suwannarut, Silvere Vialet-Chabrand, Elias Kaiser

**Affiliations:** Horticulture and Product Physiology, Wageningen University and Research, Wageningen, Netherlands

**Keywords:** C3, C4, gas exchange, fluctuating light intensity, photosynthesis, stomatal conductance

## Abstract

Photosynthesis (*A*) and stomatal conductance (*g_s_*) change diurnally due to internal signals, but the effects of diurnal rhythms on dynamic photosynthetic behavior are understudied. We examined diurnal changes in *A* and *g_s_* in ten tropical species: across species, there was a tendency for *A* and *g_s_* to decline diurnally when these were repeatedly measured under either steady-state or fluctuating irradiance conditions. We then examined in more detail the irradiance-induced kinetics of gas exchange in a C_3_ and C_4_ crop species each, namely fig (*Ficus carica*) and sugarcane (*Saccharum officinarum*). During the day, fig showed significantly slower photosynthetic induction and lower *g_s_*, as well as a slower *g_s_* increase, in the afternoon than in the morning and noon. Sugarcane showed a reduction in steady-state *A* reached under high irradiance and slower *g_s_* increase as well as lower *g_s_* reached under high irradiance, but no changes in the rate of photosynthetic induction, in the afternoon, compared to morning and noon. These reductions in the afternoon were not reverted by a dark treatment in the middle of the day, suggesting that the decrease was not proportional to diurnal time-integrated carbon fixation. Repeated exposure to light- and shadeflecks (1000 and 50 μmol m^-2^ s^-1^, lasting 20 min each) revealed fundamental differences in stomatal regulation between species: in fig, stomata opened and closed slowly, and their opening became progressively slower under a series of lightflecks, whereas sugarcane showed much faster stomatal opening than closure that was unchanged during the course of the day. Our results highlight that steady-state rates and irradiance-induced kinetics of photosynthesis and stomatal movement change diurnally in most species studied, and that they do so differently in fig and sugarcane.

## 1 Introduction

Plants continuously exchange gases with their environment. To match their metabolism with the environment, plants respond to both external and internal cues. While the effects of external drivers such as irradiance, CO_2_ concentration and temperature on photosynthetic gas exchange are apparent, internal drivers have effects as well (Matthews et al., 2017): for example, 15-25% of diurnal variation in net photosynthesis rate (*A*), 30-35% of changes in stomatal conductance ( *ɡ_s_*) during the photoperiod, and a monotonic, ~30% decline in nocturnal mitochondrial respiration, all can take place under constant environmental conditions, and are thought to be driven by several internal processes (Resco de Dios, 2017; Resco de Dios and Gessler, 2018; Bruhn et al., 2022). An implication is that purely due to internal rhythms, the efficiencies of resource use, such as irradiance and intrinsic water use efficiency (WUE_i_), change during a 24 h period (Matthews et al., 2018). However, to what extent these diurnal signals affect the dynamics of *A*, *ɡ_s_* and WUE_i_ under fluctuating solar irradiance is not well documented (Vialet-Chabrand et al., 2017b; Matthews et al., 2018). Apart from circadian rhythms, most hypotheses about the diurnal signals and processes involved (e.g. photoinhibition, photoassimilate accumulation; (Mohotti and Lawlor, 2002; Paul and Pellny, 2003) suggest that it is time-integrated and disappears during the night. This raises the question if the diurnal reductions in *A* and ɡ_s_ and changes in the speed of response could be mitigated or reversed by a period of darkness or low irradiance during the middle of the day.

More often than not, plants grow under fluctuations in irradiance that are caused by wind-induced leaf movement, passing clouds, as well as the natural rotation of the Earth (Pearcy et al., 1996; Kaiser et al., 2018; Durand et al., 2021). Photosynthesis responds to sudden increases in irradiance with a delay, which is greater the longer a leaf had been in the shade before the irradiance increase. This delay is due to processes such as enzyme activation (biochemical limitations; Ernstsen et al., 1997) and stomatal opening (diffusional limitations; Parkhurst, 1994; Lawson and Vialet-Chabrand, 2019) that limit the rapid increase in *A* (Pearcy, 1988). When summed up throughout the photoperiod, the resulting loss in foregone CO_2_ assimilation may be in the range of 10-40% (Lawson and Blatt, 2014; Long et al., 2022), and the speed with which *A* responds to fluctuations in irradiance (dynamic *A*) thus presents an exciting opportunity for crop breeding and genetic modification (Kromdijk et al., 2016; Kaiser et al., 2019). There exists large inter- and intraspecific variation in properties of dynamic photosynthesis (McAusland et al., 2016; Soleh et al., 2017; Salter et al., 2019; Zhang et al., 2022). The ability to respond to irradiance changes is often quantified using measurements of photosynthetic induction (Kaiser et al., 2017; Kaiser et al., 2019): a leaf is first adapted to darkness or shade, is then exposed to high irradiance in a stepwise change, and the resulting increase in *A* is followed until *A* reaches a steady state at high irradiance (tens of minutes). Parameters that quantify the speed of the change in *A*, such as the times to reach 50 and 90% of final steady-state (t*_A_*_50_ and t*_A_*_90_, respectively) can then be calculated for comparison between species or treatments. The extent to which genotypic variation affects the diurnal rhythms of photosynthetic gas exchange is not well documented, especially with regard to diurnal variations in dynamic photosynthesis.

One source of variation between species is the type of photosynthetic metabolism (for a review that compares the physiologies of C_3_, C_4_ and CAM plants, see Yamori et al., 2014). Whether or not C_4_ plants use fluctuating irradiance more efficiently than C_3_ plants is under discussion, but may depend on the frequency of these fluctuations: the increased complexity in the C_4_ metabolism may lead to a slower build-up of metabolite pools and incoordination between metabolic pathways, or – conversely - lead to increased flexibility that would reduce the reliance on non-photochemical quenching during the first seconds after a low to high irradiance transition (Stitt and Zhu, 2014; Slattery et al., 2018). Additionally, how the time of day affects the photosynthetic response to irradiance fluctuations in C_4_ plants, and how this compares to C_3_ plants, is currently unknown.

The aim of this study was to compare how diurnal signals influence dynamic and steady-state gas exchange in tropical plant species that have not received a lot of attention, but are economically important. We investigated i) the impact of diurnal regulation of dynamic and steady-state photosynthetic gas exchange in ten different tropical plant species, of which nine were C_3_ and one was a C_4_ species, ii) in detail the differences in dynamic *A* and g_s_ behavior throughout the day between a C_3_ and a C_4_ crop (*Ficus carica* and *Saccharum officinarum*, respectively), and iii) if the observed diurnal declines in these traits could be reversed or mitigated by an intermittent dark or low light intensity period.

## 2 Materials and methods

### 2.1 Plant materials and growth conditions

Two greenhouse compartments at Wageningen University and Research, the Netherlands (52°N, 5.5°E) constantly house a large number of tropical species, which grow in soil. All plants are irrigated with tap water by hand once or twice a week, as needed. The soil is regularly fertilized with cow manure and tree bark. Among these species, ten species were chosen based on their economic importance in tropical countries (**Table 1**; **Figure S1**). During the measurement period (June-September 2022), average day/night air temperatures and relative humidities (RH) were 24/19.4°C and 67/77%, respectively (**Table S1**). A shade screen (Ludvig Svensson, Sweden) was closed at solar incoming radiation >1075 μmol m^-2^ s^-1^ photosynthetic photon flux density (PPFD).

**Table 1:** List of investigated tropical plant species.

Common name	Scientific name	Family	Photosyn-thetic pathway	Functional type	Measured leaf^1^
American taro	*Xanthosoma sagittifolium*	Araceae	C_3_	Herbaceous	1^st^ leaf
Banana	*Musa cvs.*	Musaceae	C_3_	Herbaceous	2^nd^ leaf
Bitter wood	*Quassia amara*	Simaroubaceae	C_3_	Evergreen shrub or tree	3^rd^ leaf, 3^rd^ leaflet
Coffee (arabica)	*Coffea arabica*	Rubiaceae	C_3_	Shrubs or small tree	6^th^ leaf, 6^th^ leaflet
Fig	*Ficus carica*	Moraceae	C_3_	Deciduous shrub or small tree	6^th^ leaf
Fragrant pandan	*Pandanus amaryllifolius*	Pandanaceae	C_3_	Shrub or small tree	6^th^ leaf
Ginger	*Zingiber officinale*	Zingiberaceae	C_3_	Herbaceous perennial	3^rd^ leaf
Sugarcane	*Saccharum officinarum*	Poaceae	C_4_	Tufted perennial grass	6^th^ leaf
Tea	*Camellia sinensis*	Theaceae	C_3_	Evergreen shrub or small tree	6^th^ leaf
Yoruba soft cane	*Thaumatococcus daniellii*	Marantaceae	C_3_	Stemless herbaceous perennial	Fully mature leaves^2^

^1^Leaf position was counted from the top of the plants.

^2^All leaves extend directly from a tuber (see **Figure S1**), so leaf rank cannot be established.

### 2.2 Gas exchange measurements

Gas exchange was measured on 6 cm^2^ of the uppermost, fully expanded leaves (**Table 1**), using the Li-6800 photosynthesis system (Li-Cor Biosciences, Lincoln, NE, USA). The flow rate of air was set to 500 µmol s^-1^, the CO_2_ concentration was 400 µmol CO_2_ mol^-1^ air, and irradiance was provided by a mixture of 90% red and 10% blue LEDs in the 6800-01A fluorometer. Environmental conditions in the cuvette were controlled to roughly match average conditions in the greenhouse: 26°C air temperature and 60% RH.

#### 2.2.1 Photosynthetic induction

Plants were assessed for gas exchange responses to a step increase in irradiance from 50 to 1000 µmol m^-2^ s^-1^ (PPFD) at different times of the day: morning (9:00-10:30), noon (12:00-13:30), and afternoon (15:00-16:30). A first protocol was designed for fast screening of several species to separate fast and slow photosynthesis induction phenotypes; thereafter, a second protocol requiring lengthier but more detailed measurements was designed to study the causes of limitations in photosynthesis during photosynthetic induction. During measurements, gas exchange was recorded every second. For screening of diurnal changes in dynamic photosynthesis properties, photosynthetic induction in all ten species was measured during five min of high irradiance exposure. Leaves were covered with aluminum foil for 30 min for dark adaptation, and were then placed in the gas exchange cuvette at an irradiance of 50 µmol m^-2^ s^-1^ for five min, after which irradiance was increased to 1000 µmol m^-2^ s^-1^ for another five min. For subsequent measurements, fig and sugarcane were selected for measuring photosynthetic induction during 30 min of illumination. Leaves were enclosed in the gas exchange cuvette, exposed to 50 µmol m^-2^ s^-1^ for 35 min (during which both *A* and *ɡ_s_* reached constant values), and were then exposed to 1000 µmol m^-2^ s^-1^ for 30 min. The measurement was performed in each species with one replicate per day, until five replicates had been collected. To assess the impact of an intermittent dark period on subsequent photosynthesis, leaves were wrapped in aluminum foil for five hours (10:00-15:00), and photosynthetic induction was measured before (9:00-10:00) and after (15:00-16:30) the dark period. Environmental conditions in the greenhouse were monitored throughout the experiment (**Figure S2**).

#### 2.2.2 Steady-state measurements

To characterize gas exchange throughout the photoperiod, snapshot measurements on leaves of all ten species were conducted every 30 min between 9:00 and 16:00. During each measurement, the leaf was enclosed for ~2 min in the gas exchange cuvette at an irradiance of 200 µmol m^-2^ s^-1^, until *A* and *ɡ_s_* had stabilized, and their values were logged. The value of 200 µmol m^-2^ s^-1^ PPFD (ca. 10% of full sunlight) was chosen to represent an intermediate between full shade and full sunlight exposure. In between measurements, leaves were exposed to the greenhouse environment.

#### 2.2.3 Light- and shadeflecks

To study how fig and sugarcane leaves responded to repeated, rhythmic changes in irradiance during the day (9:00-16:00), a lightfleck protocol was devised: 50 µmol m^-2^ s^-1^ for 20 min (shadeflecks) were followed by a stepwise change to 1000 µmol m^-2^ s^-1^, which was applied for another 20 min (lightflecks). This cycle was repeated for a total of 7 hours. Gas exchange was recorded every 10 s. The measurement was performed on one replicate per day, until five replicates per species had been collected.

### 2.3 Calculations

Photosynthetic induction was calculated following Chazdon and Pearcy (1986):


(1) $$Photosynthetic\;induction\;\left(\%\right)=\;\frac{A-A_{i}}{A_{f}-\;A_{i}}\;\times 100$$

Where *A*, *A*_i,_ and *A*_f_ are transient *A* during photosynthetic induction, initial steady-state *A* at 50 µmol m^-2^ s^-1^, and final steady-state *A* at 1000 µmol m^-2^ s^-1^, respectively. Times to reach 50 and 90% of full photosynthetic induction (t*_A_*_50_, t*_A_*_90_) were calculated. In some cases, *A* showed strong overshooting behavior during photosynthetic induction (i.e., *A* at first increased to a transient peak value, and then decreased to settle on a final value); for these cases, we also calculated t*_A_*_50_ and t*_A_*_90_, using the peak value of *A* as A_f_. WUE_i_ was calculated as:


(2) $$WUE_{i}=\;\frac{A}{{ɡ}_{s}}$$

The temporal response of *ɡ_s_* to a stepwise increase and decrease in irradiance was calculated by using a curve-fitting routine in Microsoft Excel (Vialet-Chabrand et al., 2017b), which was described by sigmodal equations as follows:


(3) $${ɡ}_{s}=\left({ɡ}_{smax}-{ɡ}_{smin}\right)e^{\left(\frac{\lambda -t}{\tau_{i}}+1\right)}+\;{ɡ}_{smin}$$

(4) $${ɡ}_{s}=\left({ɡ}_{smin}-{ɡ}_{smax}\right)e^{\left(\frac{\lambda -t}{\tau_{d}}+1\right)}+\;{ɡ}_{smax}$$

where *ɡ_smax_* and *ɡ_smin_* represent the maximum and minimum steady state values of *ɡ_s_*, $\tau_{i}$ and $\tau_{d}$ represent the time constants for the increase (equation 3) and decrease (equation 4) in *ɡ_s_*, *λ* is the initial time lag, and *t* is the time at which *ɡ_s_* is calculated from time = 0. All calculations were performed per replicate, and resulting parameters were used for statistical analysis.

### 2.4 Statistical analysis

All data are shown as average ± standard error (SE), based on three to five biological replicates per treatment (n = 3 – 5). Data were initially tested for homogeneity of variances (Levene’s test) and normal distribution (Shapiro–Wilk test). On datasets where those requirements were met, one-way repeated measures ANOVA was performed, followed by Fisher’s least significant difference (LSD) procedure to determine significant differences between treatments. Paired two-sample student’s t-tests were conducted for testing for significant differences between morning and afternoon in the intermittent dark treatment experiment. When datasets did not comply with normality or homogeneity of variances, the Kruskal–Wallis test was used instead of ANOVA, followed by Dunn’s test of multiple comparisons. Then, a Mann–Whitney U-test was conducted instead of the t-test. All statistical tests were performed using RStudio v.4.1.3 (R Core Team, 2022). Statistically significant differences were determined at *p* = 0.05.

## 3 Results

### 3.1 Dynamic and steady-state photosynthesis at different times of day in ten tropical species

**Figure 1:**
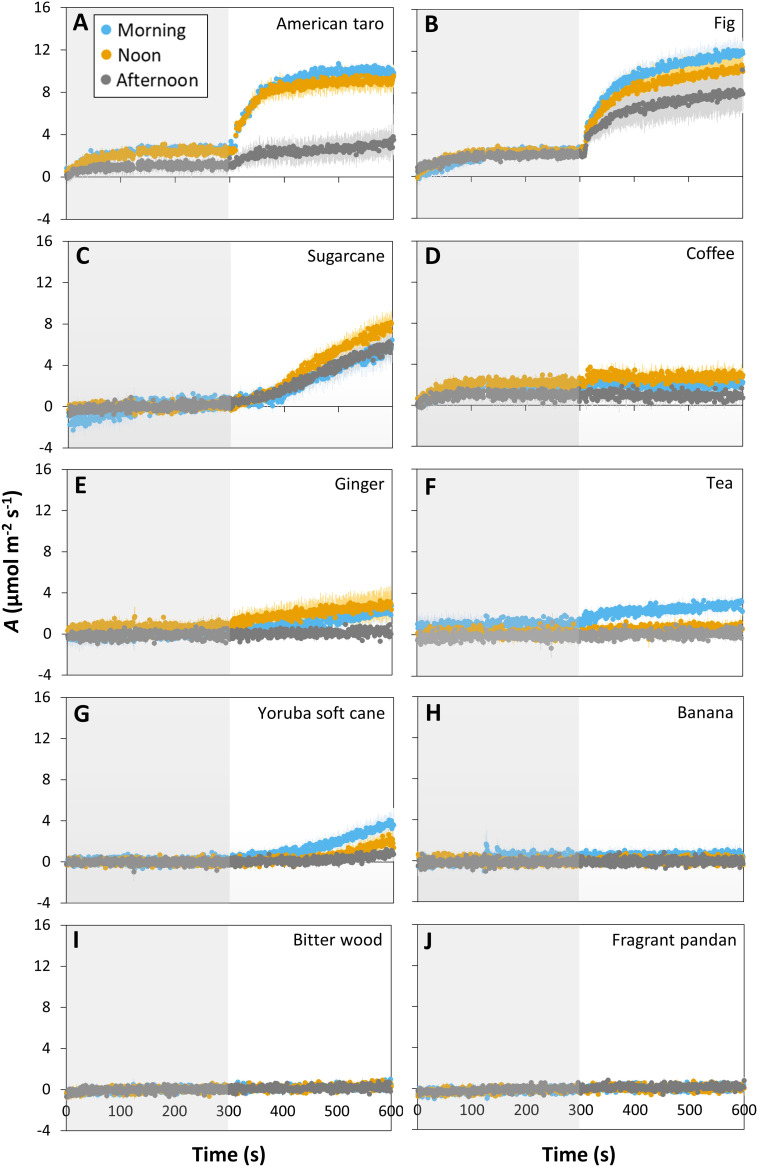
Time courses of net photosynthesis rate (*A*) in ten tropical plant species after a transition from low irradiance (50 µmol m^-2^ s^-1^, gray background) to high irradiance (1000 µmol m^-2^ s^-1^, white background) for 5 min (300 s) at different times of the day: morning (9:00-10:30), noon (12:00-13:30), and afternoon (15:00-16:30). Leaves were dark-adapted for 30 min before time = 0 s. Symbols represent averages ± SE, n = 3-5. Different panels show responses for American taro **(A)**, Fig **(B)**, Sugarcane **(C)**, Coffee **(D)**, Ginger **(E)**, Tea **(F)**, Yoruba soft cane **(G)**, Banana **(H)**, Bitter wood **(I)**, and Fragrant pandan **(J)**.

The ten tropical plant species responded very differently to a short (5 min) step change in irradiance from 50 to 1000 μmol m^-2^ s^-1^ (lightfleck; **Figure 1**): American taro and fig showed a hyperbolic increase in *A* (**Figures 1A, B**), whereas sugarcane, coffee, ginger, tea and Yoruba soft cane showed linear increases in *A* that were very small in most cases (except for sugarcane; **Figures 1C–G**). A third group, namely banana, bitter wood, and fragrant pandan, did not show any significant change in *A* (**Figures 1H–J**). The average rate of increase in *A* during the first 100 s of high irradiance exposure varied greatly between species, ranging from ~0 μmol m^-2^ s^-2^ in coffee to 0.044 μmol m^-2^ s^-2^ in fig (**Figure S3**). The ability to use a lightfleck for photosynthesis decreased during the day, but did so with very different magnitude in the studied species: American taro, for example, showed a strong (but non-significant; *p* = 0.09 based on a paired t-test) decrease in *A* in the afternoon, only reaching ~30% of the values it had reached in the morning and noon (**Figure 1A**). Fig, ginger, coffee and Yoruba soft cane showed comparatively smaller, nonsignificant decreases in *A* in the afternoon. The only species that showed a significant reduction in *A* between morning and the rest of the day was tea (*p* = 0.006; **Figure 1F**). While sugarcane, coffee and ginger tended to have larger *A* values at noon than in the morning, no species showed peak values in the afternoon.

**Figure 2:**
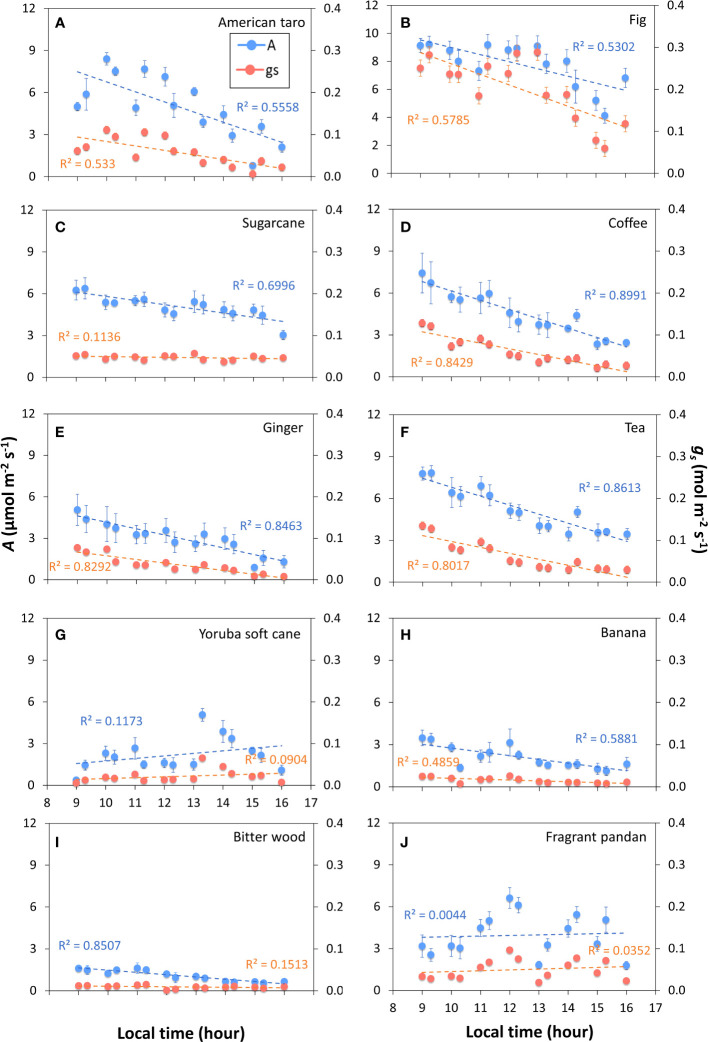
Net photosynthesis rate (*A*) and stomatal conductance (*g_s_*) throughout the photoperiod in ten tropical plant species. At every time point, measurements were conducted in snapshot-style measurements (~2 min per sample) under constant environmental conditions, whereas in between measurements, environmental conditions were variable. During measurements, irradiance was 200 μmol m^-2^ s^-1^ PPFD. Filled symbols show averages ± SE (n = 3-5), dashed lines show linear trends, R^2^ is the coefficient of determination. Different panels show responses for American taro **(A)**, Fig **(B)**, Sugarcane **(C)**, Coffee **(D)**, Ginger **(E)**, Tea **(F)**, Yoruba soft cane **(G)**, Banana **(H)**, Bitter wood **(I)**, and Fragrant pandan **(J)**.

The tendency for photosynthetic gas exchange to decline during the photoperiod also showed in most species when steady-state *A* and *ɡ_s_* were repeatedly measured at the same irradiance during the day (**Figure 2**). In Bitter wood, coffee, ginger, sugarcane, tea, American taro, banana and fig, the general trend was a monotonic decline in *A*, which was paralleled by a similar decline in *ɡ_s_* in many cases (**Figure S4**). It is noteworthy that in the C_3_ species bitter wood and in the C_4_ species sugarcane, *A* declined whereas *ɡ_s_* was relatively stable (and low) throughout the day, leading to a decline in WUE_i_ throughout the day. Contrary to the other species, fragrant pandan and Yoruba soft cane did not show clear declines, but several in- and decreases in *A* and *ɡ_s_* throughout the day (**Figures 2G, J**). In general, all species tended to show a lower value of *A* in the afternoon compared to the rest of the diurnal period.

### 3.2 Time of day effects on photosynthetic induction in fig and sugarcane

To study the diurnal response of photosynthetic gas exchange under fluctuating irradiance in greater detail, fig and sugarcane were chosen, because both species i) showed large changes in *A* and *ɡ_s_* throughout the day (**Figures 1**, **2**), ii) use different photosynthesis pathways, C_3_ and C_4_ respectively, iii) have kidney- and dumbbell-shaped stomata, respectively, and iv) are commercially relevant crops.

**Figure 3:**
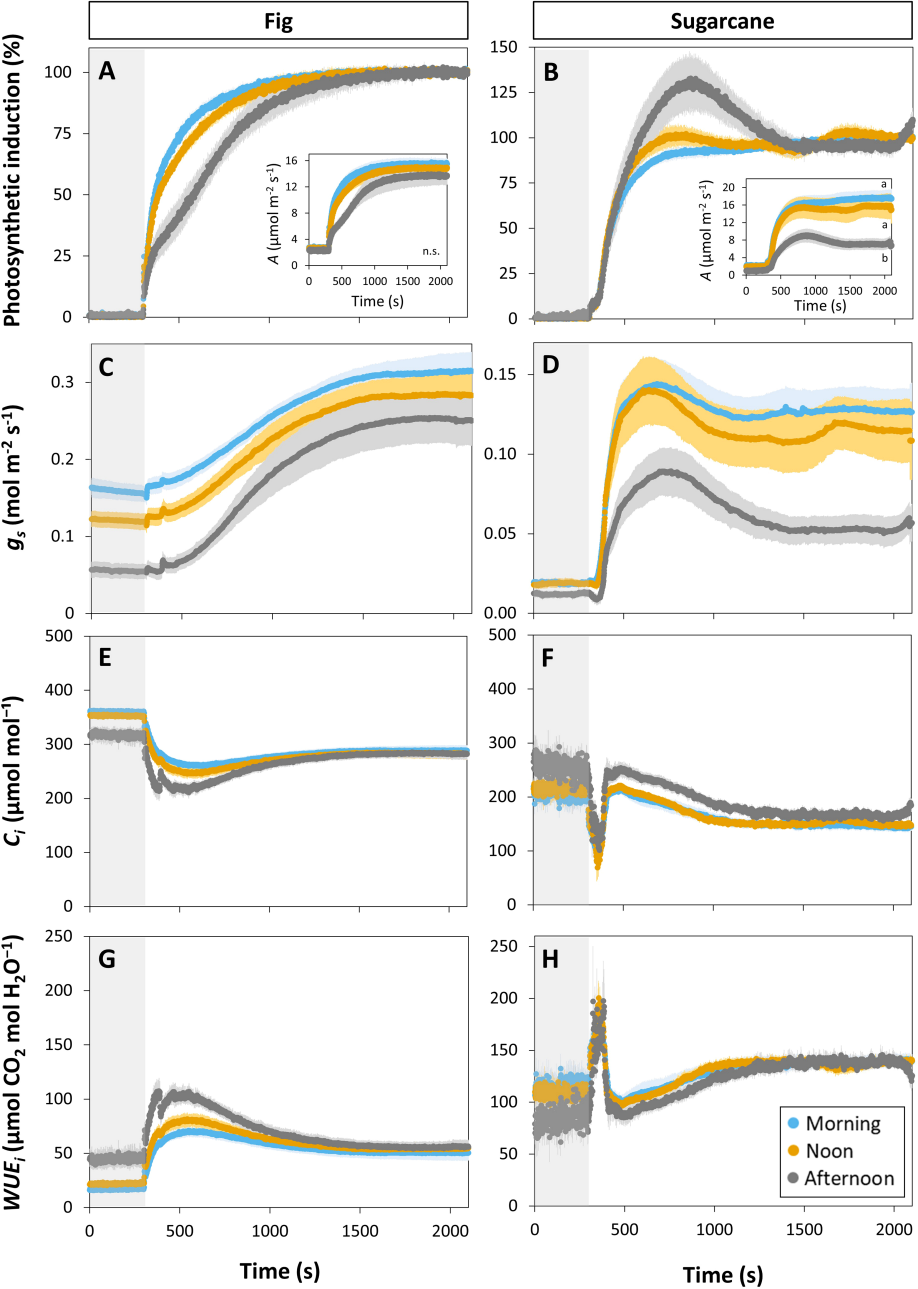
Time courses of gas exchange in fig (C_3_, left panel) and sugarcane (C_4_, right panel) after a transition from low (50 µmol m^-2^ s^-1^, gray background) to high irradiance (1000 µmol m^-2^ s^-1^) for 30 min (1800 s) at different times of the day: morning (9:00-10:30), noon (12:00-13:30), and afternoon (15:00-16:30). Before transition to high irradiance, leaves were adapted to 50 µmol m^-2^ s^-1^ for 35 min. **(A, B)**, photosynthetic induction; **(C, D)**, stomatal conductance (*g_s_*); **(E, F)**, CO_2_ concentration in the substomatal cavity (C_i_); **(G, H)**, intrinsic water use efficiency (WUE_i_). Insets in **(A, B)** show time courses of net photosynthesis rate (*A*). Symbols represent averages ± SE, n = 5. Note that y-axis scales are different between plots.

**Figure 4:**
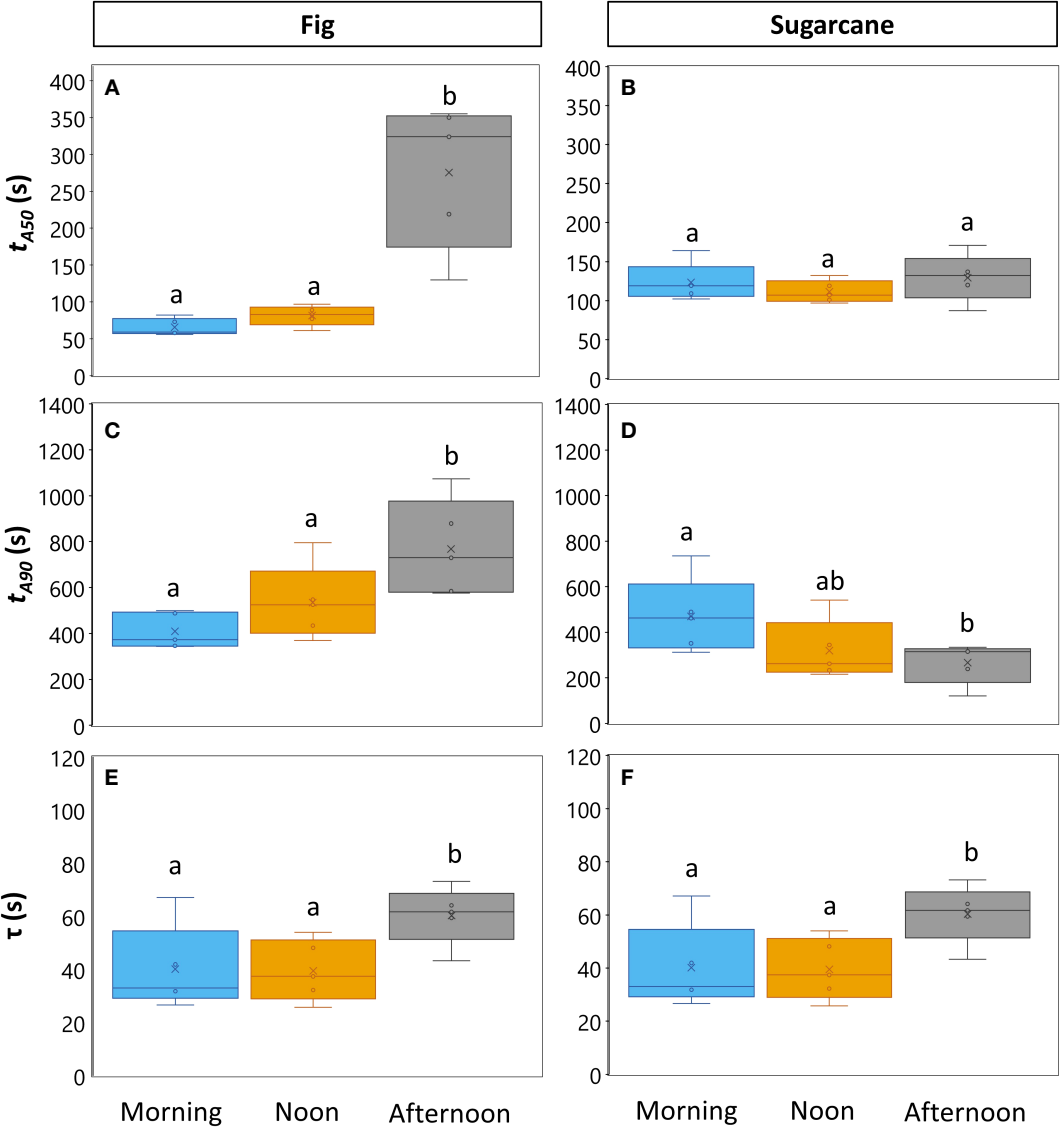
Parameters expressing speed of photosynthesis and stomatal conductance responses to an irradiance change in fig (C_3_, left panel) and sugarcane (C_4_, right panel). For details on experiment, see **Figure 3**. **(A-D)**. Times required to reach 50% (**A, B**; t*_A_*_50_) and 90% (**C, D**; t*_A_*_90_) of final steady-state net photosynthesis rate during photosynthetic induction; **(E, F)**, time constant of stomatal opening. Different letters above boxplots indicate statistically significant differences between times of day (*p*<0.05), as determined by Fisher’s LSD method. The vertical line inside the boxplot represents the median, the x represents the average value, upper and lower limits to the box represent the first and third quantile, respectively, and the whiskers represent the minimum and maximum values. Note that y-axis scales are different between plots.

Photosynthesis and stomatal conductance reached after 30 min under high irradiance decreased during the diurnal period (**Figure 3**), similarly to observations in **Figures 1** and **2**. In addition, the temporal kinetics of *A* and *ɡ_s_* showed species-specific differences throughout the day (**Figures 3**, **4**). In fig, the rate of photosynthetic induction declined throughout the day (**Figures 3A**; **S5A**), resulting in significantly larger values of t*_A_*_50_ and t*_A_*_90_ in the afternoon compared to morning and noon (**Figures 4A, C**). This decline was paralleled by a gradual reduction in *ɡ_s_* (**Figure 3C**), slower stomatal opening in the afternoon (larger *τ*, **Figure 4E**) resulting in a larger reduction in C_i_ during initial phases of photosynthetic induction (**Figure 3E**), and a tendency for WUE_i_ to increase (**Figure 3G**). Despite this decrease in the rate of photosynthetic induction in fig, steady-state *A* at high irradiance was similar between different times of day (**Figure 3A**, inset). In sugarcane, on the other hand, the rate of photosynthetic induction did not change much throughout the day (**Figures 3B**, **4B, D**), while steady-state *A* at the end of the irradiance phase dropped by more than half in the afternoon, reaching a significantly lower level compared to the other two time points (**Figure 3B**, inset). When t*_A_*_50_ and t*_A_*_90_ were calculated based on the peak value of *A* reached during photosynthetic induction (to account for the overshooting behavior of *A*), it could still be concluded that the rate of photosynthetic induction was not affected much in sugarcane by the time of day (**Figure S6**). Also, although photosynthetic induction was not strongly different between times of day in sugarcane (**Figures 4B, D**; **S6**), the absolute rate of increase in *A* did slow down significantly in the afternoon (*p* = 0.02; **Figure S5B**).

The decrease in steady-state *A* was paralleled by a decrease in steady-state *ɡ_s_* at high irradiance, which in the afternoon was reduced by more than half (compared to noon and morning; **Figure 3D**). The temporal kinetics of *ɡ_s_* mostly differed by their decreasing levels (*ɡ_smin_* and *ɡ_smax_*) at different times of the day rather than their shape in fig, but were lower (*ɡ_smax_*) and slower in the case of sugarcane (**Figure 4F**). Further, *ɡ_s_* in sugarcane exhibited overshooting behavior, as well as a reduction after the overshoot, both of which tended to get stronger as the day progressed (**Figure 3D**); this was coordinated with *A* responses, leading to strong transient increases and drops in *A* until a steady-state value was reached (**Figure 3B**). In sugarcane, C_i_ was low and WUE_i_ high, both staying relatively unchanged throughout the day (**Figures 3F, H**) compared to fig, as would be expected of a C_4_ when compared to a C_3_ species.

**Figure 5:**
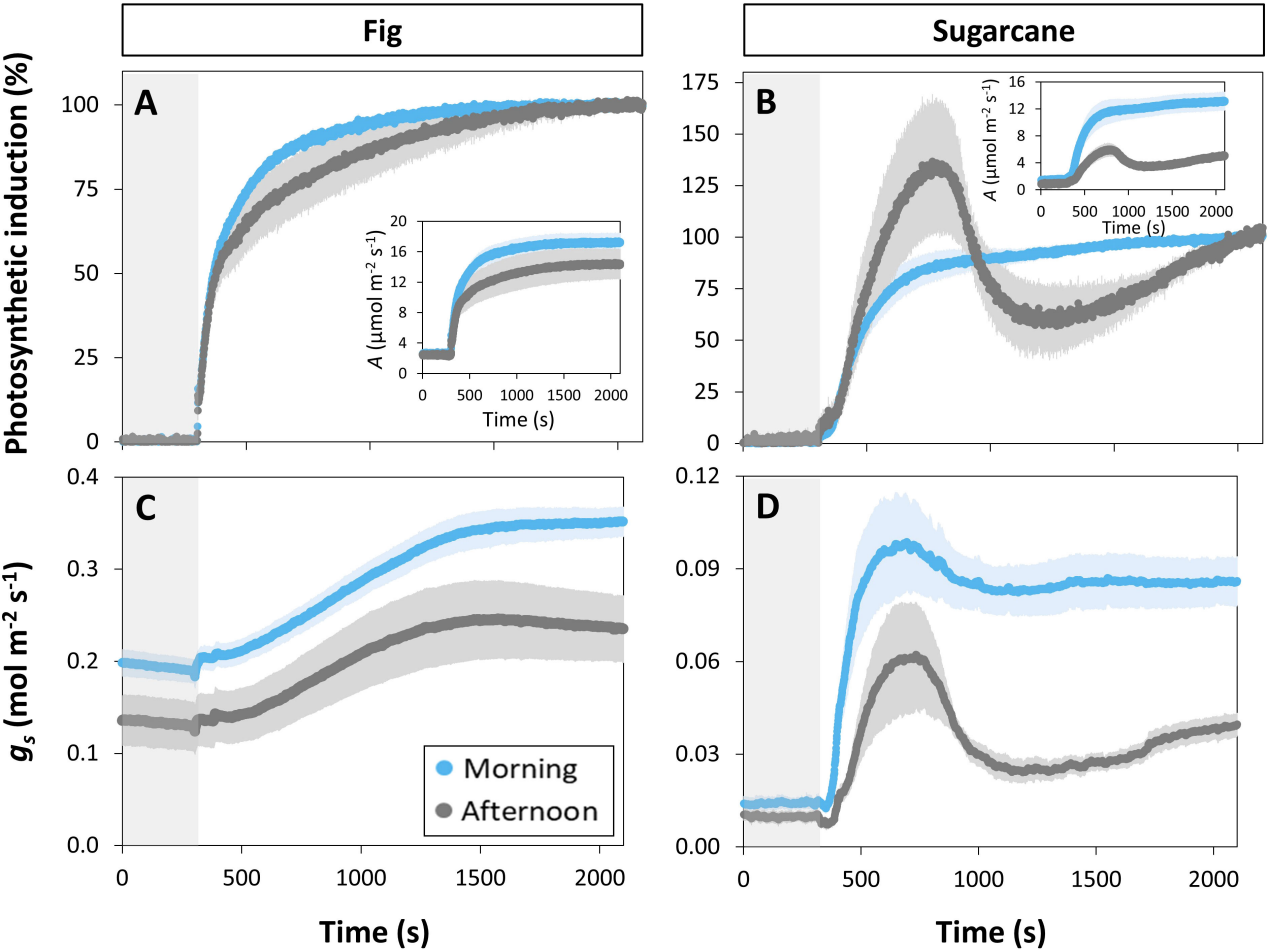
Results of midday dark adaptation experiment. Time courses of gas exchange in fig (C_3_, left panel) and sugarcane (C_4_, right panel) after a transition from low (50 µmol m^-2^ s^-1^, gray background) to high irradiance (1000 µmol m^-2^ s^-1^) for 30 min (1800 s) in the morning (9:00-10:00), and in the afternoon (15:00-16:30) after intermittent dark adaptation for five hours (10:00-15:00). Before transition to high irradiance, leaves were adapted to 50 µmol m^-2^ s^-1^ for 35 min. **(A, B)**, photosynthetic induction; **(C, D)**, stomatal conductance (*g_s_*). Insets in **(A, B)** show time courses of net photosynthesis rate (*A*). Symbols represent averages ± SE, n = 5. Note that y-axis scales are different between plots.

Next, we wanted to know whether the diurnal decline of photosynthesis and stomatal conductance traits was caused by total irradiance exposure during the photoperiod, and consequently could be alleviated by a dark period (applied between 10:00 and 15:00). This seemed not to be the case (**Figures 5**; **S7**), as i) *A* and *ɡ_s_* in sugarcane still showed the same strong decline (**Figures 5B, D**) and overshooting behavior they had shown without intermittent dark treatment (**Figures 3B, D**), ii) there was still a tendency for photosynthetic induction to be slower in the afternoon (**Figure 5A**), although the difference was not significant (**Figure S7A**), and iii) *ɡ_s_* in fig still showed the same decrease as it had without intermittent dark treatment (compare **Figures 5C**, **3C**). Calculating t*_A_*_50_ and t*_A_*_90_ based on overshooting behavior in sugarcane did not change these conclusions, as there was no difference between different times of day in sugarcane regardless of how t*_A_*_50_ and t*_A_*_90_ were calculated (cv. **Figures S7**, **S8**).

### 3.3 Gas exchange during repeated light- and shadeflecks

**Figure 6:**
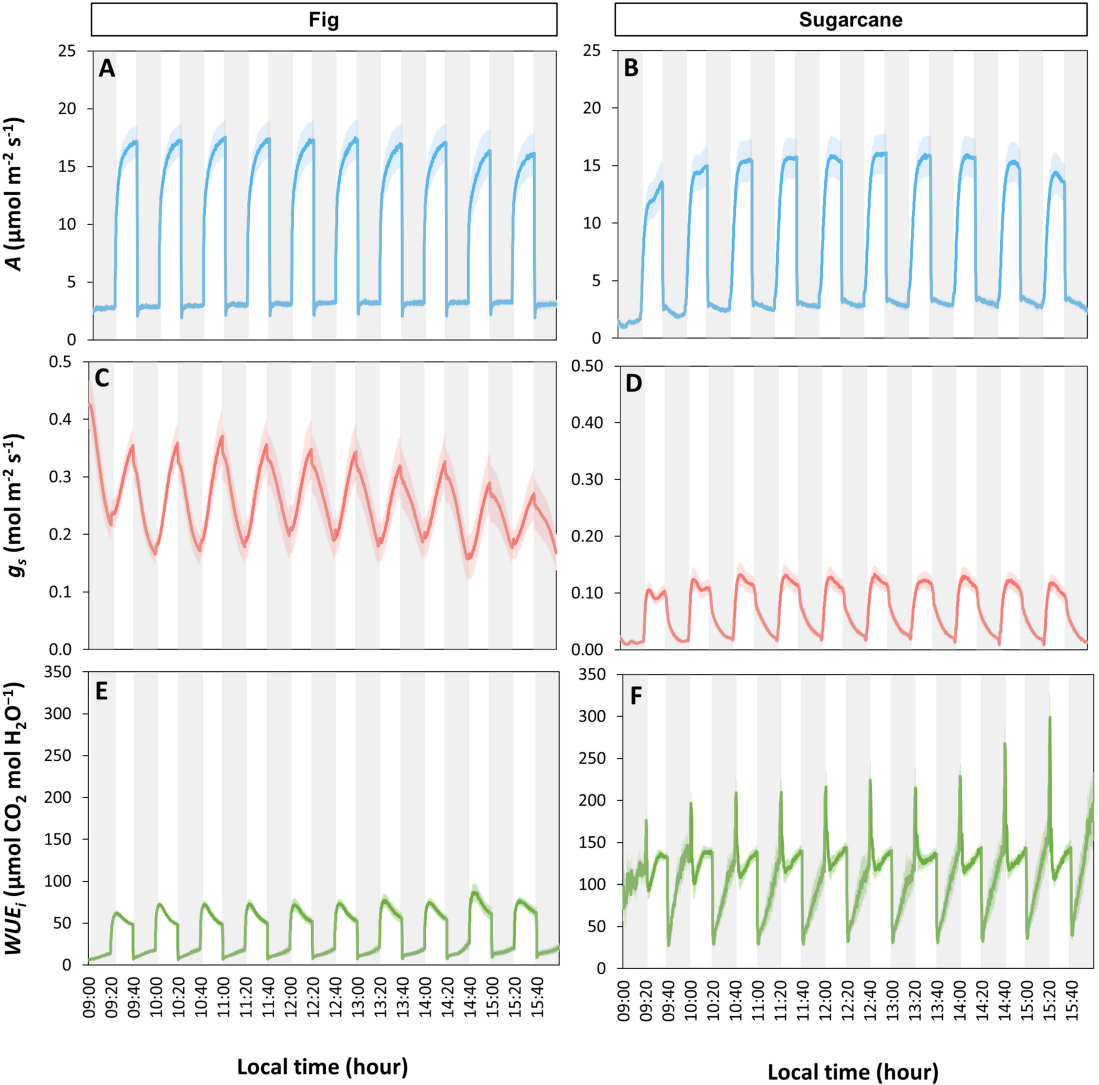
Time courses of photosynthetic gas exchange under a series of light- and shadeflecks. Leaves of fig (left panel) and sugarcane (right panel) were exposed to repeated changes between periods of low (50 µmol m^-2^ s^-1^, gray background) and high irradiance (1000 µmol m^-2^ s^-1^, white background), each period lasting 20 min, for a total of seven hours. **(A, B)**, net photosynthesis rate (*A*); **(C, D)**, stomatal conductance (*g_s_*), **(E, F)**, intrinsic water use efficiency (WUE_i_). Lines represent *a*verages ± SE, n = 5.

Next, we studied if an accumulation of irradiance fluctuations over the course of the day was the reason for the observed decrease in *A* and *ɡ_s_*. To simulate these, leaves were exposed to repeated light- and shadeflecks, i.e. a 7 h sequence of transitions between 50 and 1000 μmol m^-2^ s^-1^, each taking 20 min. In fig, *A* during lightflecks never reached a steady state, but the highest attained value tended to show a decline (**Figure 6A**), which however was not significant (*p* = 0.129; average *A* during the last 30 s of a lightfleck was compared between the first and last lightfleck). During shadeflecks, *A* was unchanged in fig throughout the day, and showed a consistent, rapid decrease and slow re-increase (post-illumination CO_2_ burst) right after the high to low irradiance transition (from ~2 to ~3 μmol m^-2^ s^-1^; **Figure 6A**). Stomatal conductance in fig displayed symmetrical in- and decreases during light- and shadeflecks, respectively (**Figure 6C**). Changes in *ɡ_s_* tended to become smaller in amplitude during the diurnal period, leading to a decrease in *ɡ_s_* (**Figure 6C**). The overall decline in *ɡ_s_* was relatively stronger than that of *A*, resulting in a tendency for WUE_i_ to increase during the day (**Figure 6E**).

Unlike in fig, *A* in sugarcane often reached a steady state during lightflecks, but in some cases showed a subsequent decline (**Figure 6B**) that coincided with declines in *ɡ_s_* (**Figure 6D**). During shadeflecks, *A* declined near-monotonically, but showed an increasing trend during the diurnal period. No post-illumination CO_2_ burst upon high to low irradiance transitions was observed in sugarcane (**Figure 6B**). Increases in *ɡ_s_* during lightflecks were extremely rapid compared to fig, but after reaching an initial peak within 5-10 min at relatively low values (ca. 0.1 mol m^-2^ s^-1^), *ɡ_s_* decreased (**Figure 6D**). Unlike the rapid *ɡ_s_* increase, the decrease in *ɡ_s_* upon high to low irradiance transitions displayed a first rapid decrease, followed by a slow exponential decay (**Figure 6D**). WUE_i_ in sugarcane was ~ 2 - 3x higher than in fig and showed complex kinetics, with large spikes in the beginning of a lightfleck and large drops at the beginning of a shadefleck, as well as strong increases during the remainder of the shadefleck, as *g_s_* decreased (**Figure 6F**).

**Figure 7:**
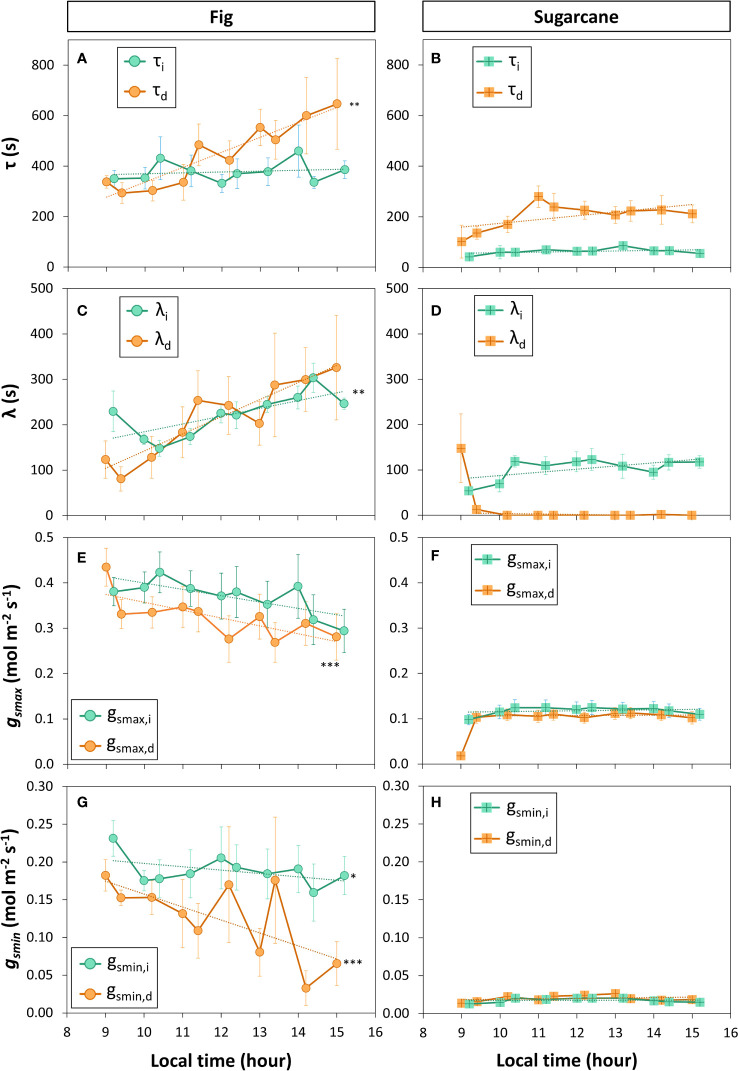
Parameters of stomatal conductance kinetics derived from a sequence of increased (X_i_) and decreased (X_d_) irradiance. Leaves of fig (left panel) and sugarcane (right panel) were exposed to repeated shadeflecks (50 µmol m^-2^ s^-1^) and lightflecks (1000 µmol m^-2^ s^-1^) for 20 min each, see **Figure 6** for details. **(A, B)**, time constant of stomatal movement (*τ*); **(C, D)**, initial lag time of stomatal movement (λ), **(E, F)**, maximum stomatal conductance (*g_smax_*); **(G, H)**, minimum stomatal conductance reached during low irradiance (g_smin_). Filled symbols show averages ± SE (n = 3-5). Stars show that the last value in the time series is significantly different from the first value, i.e. that a significant change in a given parameter has occurred during the time series; *, *p*<0.05; **, *p*<0.01; ***, *p*<0.001. Dashed lines show linear trends (in the case of λ_d_ **(D)** and g_smax,d_ **(F)**, the first time point was omitted in the linear correlation analysis).

A closer look at *ɡ_s_* kinetics during light- and shadeflecks (**Figures 6C, D**) revealed fundamental differences in stomatal regulation between the two species (**Figure 7**). Overall, fig displayed slow stomatal movement (large time constants and initial lag times; **Figures 7A, C**), as well large *ɡ_smax_* and *ɡ_smin_* (**Figures 7E, G**), but also showed a diurnal trend: the time constant for stomatal closure (*τ*_d_) increased strongly and significantly with time, showing that at the end of the measuring period stomata took ca. twice as long to close as they had during its beginning, whereas the time constant for stomatal opening (*τ*_i_) did not change with time (**Figure 7A**). Also, the lag time for stomatal opening or closure showed near-linear increases in fig (**Figure 7C**), whereas overall *ɡ_s_* (*ɡ_smax_* and *ɡ_smin_*; **Figures 7E, G**) tended to decline over time. In contrast, sugarcane showed much faster stomatal movement, as illustrated by time constants that were ca. 2-6x smaller than in fig (**Figure 7B**). It is also noteworthy that in sugarcane, stomata closed much more slowly than they opened (ca. 4x larger *τ*_d_ than *τ*_I_, **Figure 7B**), whereas such an obvious difference between the two time constants was not visible in fig (**Figure 7A**). Also, while the time lag for stomatal opening (λ_i_) was ca. half as large in sugarcane compared to fig, there was no measurable time lag for stomatal closure in sugarcane (apart from the first 1-2 shadefleck instances; **Figure 7D**). Additionally, in sugarcane overall *ɡ_s_* (*ɡ_smax_* and *ɡ_smin_*; **Figures 7F, H**) was very low and stable over time.

## 4 Discussion

Dynamic gas exchange has been shown to be an important trait to improve crop yield (Adachi et al., 2019). Our results highlight the largely different strategies deployed by tropical species to dynamically balance the trade-off between carbon fixation and stomatal control of transpiration under light intensity fluctuations. These differences were particularly striking in the C_3_ crop fig and the C_4_ crop sugarcane, where our results revealed very different and complex diurnal patterns of WUE_i_ under a succession of sunflecks.

### 4.1 Dynamic and steady-state photosynthesis and stomatal conductance tend to decline throughout the day

In most species that we surveyed, steady-state *A* and *ɡ_s_* (when repeatedly measured under stable environmental conditions) declined throughout the photoperiod (**Figures 1**–**3**). We observed a strong coupling between *A* and *ɡ_s_* in most species (**Figure S4**); however, on the basis of our data we cannot conclude whether a reduction in *g_s_* caused a reduction in *A*, or vice versa. Similarly, in the seven species that did show a transient increase in *A* during a 5 min exposure to high irradiance, there tended to be a reduction of *A* reached during afternoon measurements (**Figure 1**). Steady-state *A* and *ɡ_s_* (or transpiration rate) have been found to decline throughout the day under constant environmental conditions in e.g. Arabidopsis (*Arabidopsis thaliana;* Matthews et al., 2018), lisianthus (*Eustoma grandiflorum*; Lanoue et al., 2017), tomato (*Solanum lycopersicum*; Lanoue et al., 2017; Lanoue et al., 2018), broad bean (*Vicia faba*; Gorton et al., 1993), common bean (*Phaseolus vulgaris*, Mencuccini et al., 2000) and cotton (*Gossypium hirsutum*; Resco de Dios et al., 2017). Under fluctuating irradiance, *A* and *g_s_* have also been shown to decrease during the diurnal period in *Arabidopsis thaliana*, with a reduction of about 20% of the diurnal carbon gain (Vialet-Chabrand et al., 2017a).

During the later stages of a photoperiod, *A* (and *ɡ_s_*) may decrease due to at least two kinds of inhibition: photoinhibition and feedback inhibition. Photoinhibition is incurred when a central pigment in the photosystem II core, D1, is destroyed by oxidative stress more quickly than it can be repaired (Long and Humphries, 1994). The risk of oxidative stress in photosynthetic light harvesting antennae increases with the level of irradiance, and photosynthetic organisms have evolved a number of photoprotective processes, which aim at avoiding oxidative stress through fast non-damaging relaxation of pigment exited states, interception of reactive oxygen species through antioxidants, and repair of damaged proteins (Bassi and Dall’osto, 2021). Nevertheless, the risk of photoinhibition increases at high irradiance, and can lead to reductions of *A* (Murchie and Niyogi, 2011). Feedback inhibition is incurred when metabolites (e.g. sucrose, starch and amino acids) produced in the leaf induce a feedback regulation of photosynthesis as a two-way process (Paul and Pellny, 2003; Henry et al., 2020). A progressive accumulation of sucrose in the leaf during the photoperiod is often observed and could be caused by a limited rate of export; sucrose concentration is sensed by trehalose-6-phosphate, which subsequently triggers a reduction in *A*, among others (Figueroa and Lunn, 2016; Paul et al., 2018). We hypothesized that a dark period of several hours in the middle of the diurnal period would lead to a reduction in metabolite concentration and a repair of potentially damaged photosystem II. Under this assumption, if one of these processes was involved in the diurnal regulation of photosynthesis, its speed of response and magnitude of change should have shown a similar response at the beginning and end of the diurnal period. Our results suggest that these processes are not likely to be involved in the diurnal regulation of photosynthesis, as the dark period did not alter the response (**Figures 5**; **S7**). However, we did not quantify carbohydrate accumulation nor the degree of photoinhibition throughout the photoperiod, and more direct experimental evidence is thus needed to make a more definitive statement.

Two other possibilities for the observed reductions in *A* and *ɡ_s_* may be: regulation by the circadian clock, and changes in environmental conditions in the experimental greenhouse. Large diurnal changes in *A* and *ɡ_s_* under perfectly constant environmental conditions are known to occur (Sayre, 1926; Mansfield and Heath, 1964; Gorton et al., 1989; Resco de Dios, 2017; Matthews et al., 2018) and these can be caused by the circadian clock. To our knowledge, a separation of the effects of feedback inhibition and the circadian clock on diurnal changes in *A* and *ɡ_s_* has not been attempted. Further, the climate in the greenhouse where the measurements were conducted was not stable diurnally, but changed as a function of incoming solar irradiation: both air temperature and VPD showed peaks around mid-day on several days of measurement (**Figure S2**), likely changing whole-plant water potential. This may have had systemic effects on measurement leaves, progressively reducing *ɡ_s_* throughout the day and feeding back on *A* (Devireddy et al., 2020; Ehonen et al., 2020).

As for diurnal changes in the properties of dynamic *A*, our results suggest that in many species, the speed of photosynthetic induction decreased as the day progressed (**Figures 1**, **3**, **5**), though the cause was not clearly identified. A possible explanation could be that the lower observed *ɡ_s_* in the afternoon caused larger diffusional limitations, limiting the rate of *A* increase. The decrease in speed and magnitude of response in *ɡ_s_* during the diurnal period is likely under control of the circadian clock (Gorton et al., 1993; Vialet-Chabrand et al., 2021), as well as starch metabolism (Westgeest et al., 2023). The decrease in photosynthesis induction during the diurnal period was species-specific, but present in both C_3_ and C_4_ plants (**Figure 1**). These results are in agreement with previous findings (Poorter and Oberbauer, 1993; Allen and Pearcy, 2000a; Allen and Pearcy, 2000b; Matthews et al., 2018). One potential cause for diurnal changes in photosynthetic induction could be diurnal changes in the ratio of Rubisco to Rubisco activase (the latter of which is required for fast photosynthetic induction (Mott and Woodrow, 2000)): in wheat leaves, it was recently found that while the concentration of Rubisco activase oscillated throughout the photoperiod due to changes in gene expression, the concentration of Rubisco was stable (Perdomo et al., 2021). It could be hypothesized that a diurnal change in the ratio of Rubisco to its chaperone would lead to changes in the rate of photosynthetic induction.

### 4.2 Kinetics of stomatal movement under fluctuating irradiance differ widely between fig and sugarcane

Stomata of sugarcane responded much more quickly, and also with much smaller diurnal variation in time constants, to irradiance fluctuations than those of fig (**Figures 6**, **7**). The difference in speed of response between sugarcane and fig could be in part explained by the difference in stomatal anatomy, with subsidiary cells in sugarcane stomata (dumbbell shape; (Wang and Chen, 2020) providing a mechanical advantage (Franks and Farquhar, 2007) and more rapid responses compared to stomata in fig (kidney shape; Mamoucha et al., 2016). Our results are well in agreement with those of McAusland et al. (2016) and Ozeki et al. (2022). In McAusland et al. (2016), the three C_4_ species maize, sorghum and miscanthus (*Zea mays*, *Sorghum bicolor* and *Miscanthus nepalensis*, respectively) showed much smaller time constants of stomatal opening and closure than 12 other C_3_ species, after single step changes in irradiance. Ozeki et al. (2022) showed that whole-plant *ɡ_s_* in several C_4_ species belonging to the Poaceae family (sorghum, maize, *Eleusine coracana*, *Panicum miliaceum*, and *Zea nicaraguensis*) responded much more quickly, and with a smaller diurnal variation in time constants, to a series of 15 min and 30 min lightflecks, than did several C_3_ species (*Triticum aestivum*, *Avena sativa*, *Hordeum vulgare*, and *Lolium multiflorum*). Adding up these results, we can conclude that the kinetics of *ɡ_s_* in C_4_ leaves i) are often faster than in C_3_ leaves, unlike the kinetics of *A* (see next paragraph), and these faster ɡ_s_ kinetics are likely caused by the presence of dumbbell-shaped guard cells that are paired with subsidiary cells, and ii) show a different pattern of diurnal variation for *A* and *ɡ_s_*, unlike those of C_3_. The combination of low *ɡ_s_* and fast stomatal movement under irradiance fluctuations results in much higher dynamic WUE_i_ in C_4_ than C_3_ crops (**Figures 3**, **6**; Ozeki et al., 2022). Improving on the speed of stomatal movement through breeding or genetic manipulation is arguably a worthwhile target (Lawson and Blatt, 2014), given agricultural freshwater use and (projected future) freshwater scarcity.

### 4.3 Does C_4_ photosynthesis react faster to fluctuating irradiance than C_3_ photosynthesis?

While research on dynamic *A* had until recently largely been focused on C_3_ species, several recent studies compared dynamic *A* in a large number of C_3_ and C_4_ species (McAusland et al., 2016; Li et al., 2021; Lee et al., 2022; Ozeki et al., 2022; Arce Cubas et al., 2023). The question whether C_4_ photosynthesis uses irradiance fluctuations with higher efficiency than C_3_ photosynthesis has been discussed repeatedly (Stitt and Zhu, 2014; Slattery et al., 2018), but without a definitive conclusion. Our results did not show a significant difference in photosynthesis induction time between fig and sugarcane, suggesting that such a trait is species-specific and does not only depend on the photosynthesis pathway. Comparing the results from recent experimental papers, no clear difference between C_3_ and C_4_ species can be identified: whereas Lee et al. (2022) found that C_4_ species used a series of short lightflecks (4 min high irradiance, 2 min low irradiance) more efficiently than C_3_ species, Li et al. (2021) found the opposite to be true when exposing leaves to repeated cycles of high and low irradiance (each cycle lasting 2 min). Furthermore, Arce Cubas et al. (2023) found *A* in dark-adapted C_4_ leaves to respond more slowly to a sudden step change to either 600 or 1500 μmol m^-2^ s^-1^. Finally, data from McAusland et al. (2016) and Ozeki et al. (2022) show no obvious differences in time constants of *A* after an increase in irradiance, similar to our own results (**Figure 4**). These results suggest that there is no consistent difference in dynamic *A* between C_3_ and C_4_ plants. However, we note that i) all studies cited here use different measuring protocols of dynamic *A* (as is often the case), and ii) the hypothesis that the extent to which C_4_ leaves can utilize irradiance fluctuations depends on the frequency of these fluctuations (Slattery et al., 2018) still remains to be tested.

### 4.4 Conclusions

It is now well accepted that photosynthesis often operates under dynamically changing irradiance. However, how strongly the response of photosynthesis to irradiance fluctuations is affected by the time of day, and how this differs between species, is understudied. Hence, very little is known about the diurnal behavior of dynamic photosynthesis and stomatal conductance in plants, including various tropical plant species. Our study adds significant knowledge, in that we found that i) steady-state and dynamic photosynthesis traits tended to decline throughout the day in seven out of ten species (**Figures 1**, **2**), ii) compared to measurements in the morning and noon, in the afternoon the rate of photosynthetic induction was reduced in fig, whereas in sugarcane the steady-state photosynthesis rate was reduced (**Figures 3**, **4**), surprisingly, neither of these reductions could be reversed by intermittent dark adaptation during the day (**Figure 5**), and iii) stomata in fig opened and closed slowly, and their opening became progressively slower under a series of lightflecks, whereas sugarcane consistently showed much faster opening than closure (**Figures 6**, **7**). Overall, the diurnal gas exchange regulation is species-specific and largely impacts diurnal water use efficiency.

## Data availability statement

The original contributions presented in the study are included in the article/**Supplementary Material**. Further inquiries can be directed to the corresponding author.

## Author contributions

WS: Data curation, Formal Analysis, Methodology, Writing – original draft, Writing – review & editing. SV-C: Methodology, Supervision, Writing – review & editing. EK: Conceptualization, Methodology, Supervision, Writing – original draft, Writing – review & editing.
