# Supplementary material for: Diurnal decline in photosynthesis and stomatal conductance in several tropical species
Source: Front Plant Sci. 2023 Oct 24;14:1273802. doi: 10.3389/fpls.2023.1273802 (PMC10628437; doi:10.3389/fpls.2023.1273802)
Supplement: Supplementary file 1 [file DataSheet_1.docx]

Supplementary material to:

Suwannarut et al.: Diurnal decline in photosynthesis and stomatal conductance in several tropical species

**Table S1.** Monthly average temperature and relative humidity in the greenhouse during measurement period (June-September 2022)

| Month | June | July | August | September |
| --- | --- | --- | --- | --- |
| Average temperature (°C) | | | | |
| Day | 23.3 | 24.2 | 25.6 | 22.4 |
| Night | 18.5 | 19.9 | 21.0 | 18.2 |
| Average relative humidity (%) | | | | |
| Day | 63.9 | 71.6 | 64.6 | 67.5 |
| Night | 77.7 | 79.0 | 75.2 | 74.6 |


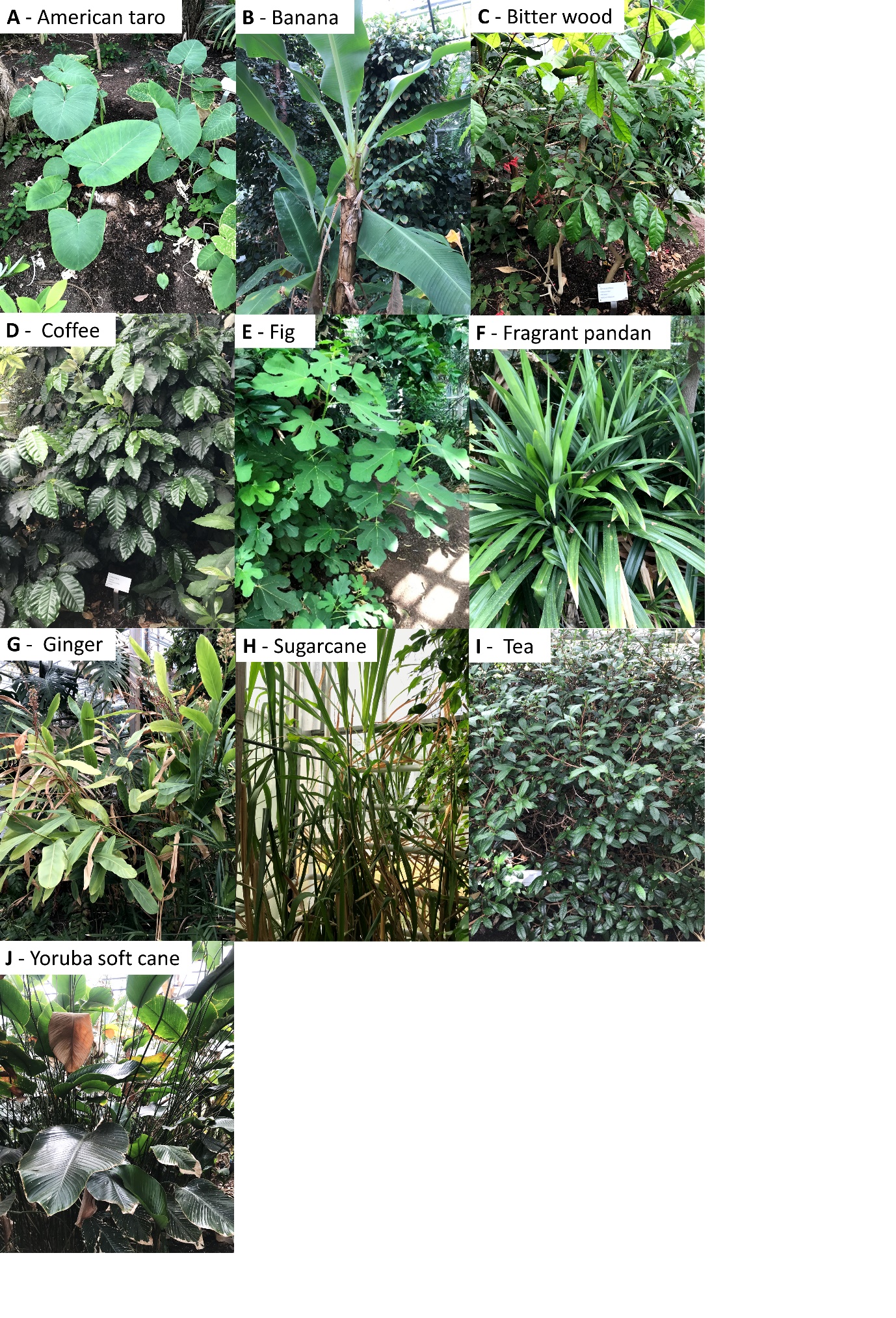


**Figure S1.** Representative pictures of all ten tropical plant species growing in the experimental greenhouse.


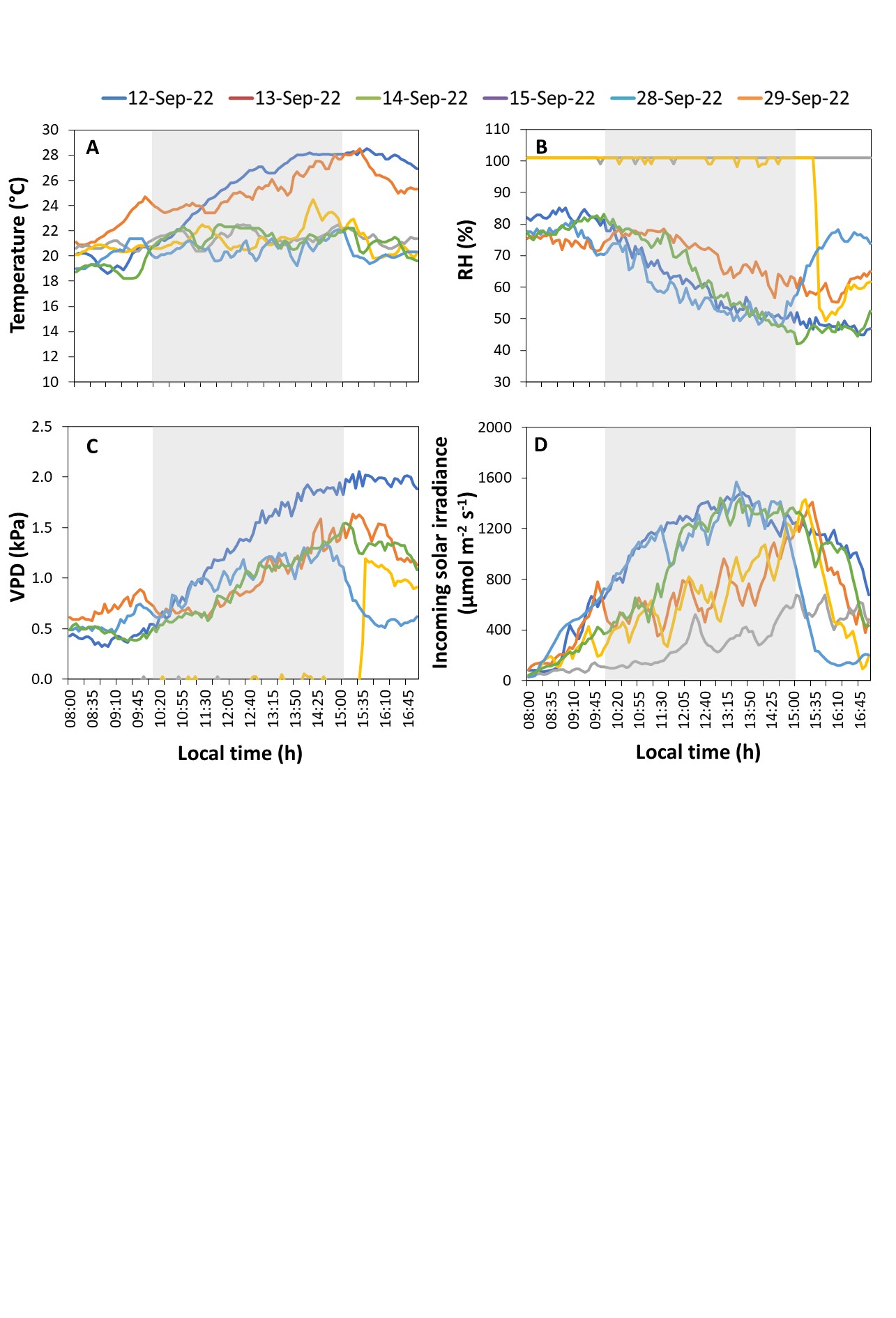


**Figure S2.** Aerial climate in the experimental greenhouse compartment during the dark adaptation experiment. A) air temperature; B) relative humidity (RH); C) vapour pressure deficit (VPD); and D) incoming solar irradiance (i.e. irradiance outside of the greenhouse). The shaded area represents the duration of dark adaptation of single leaves.


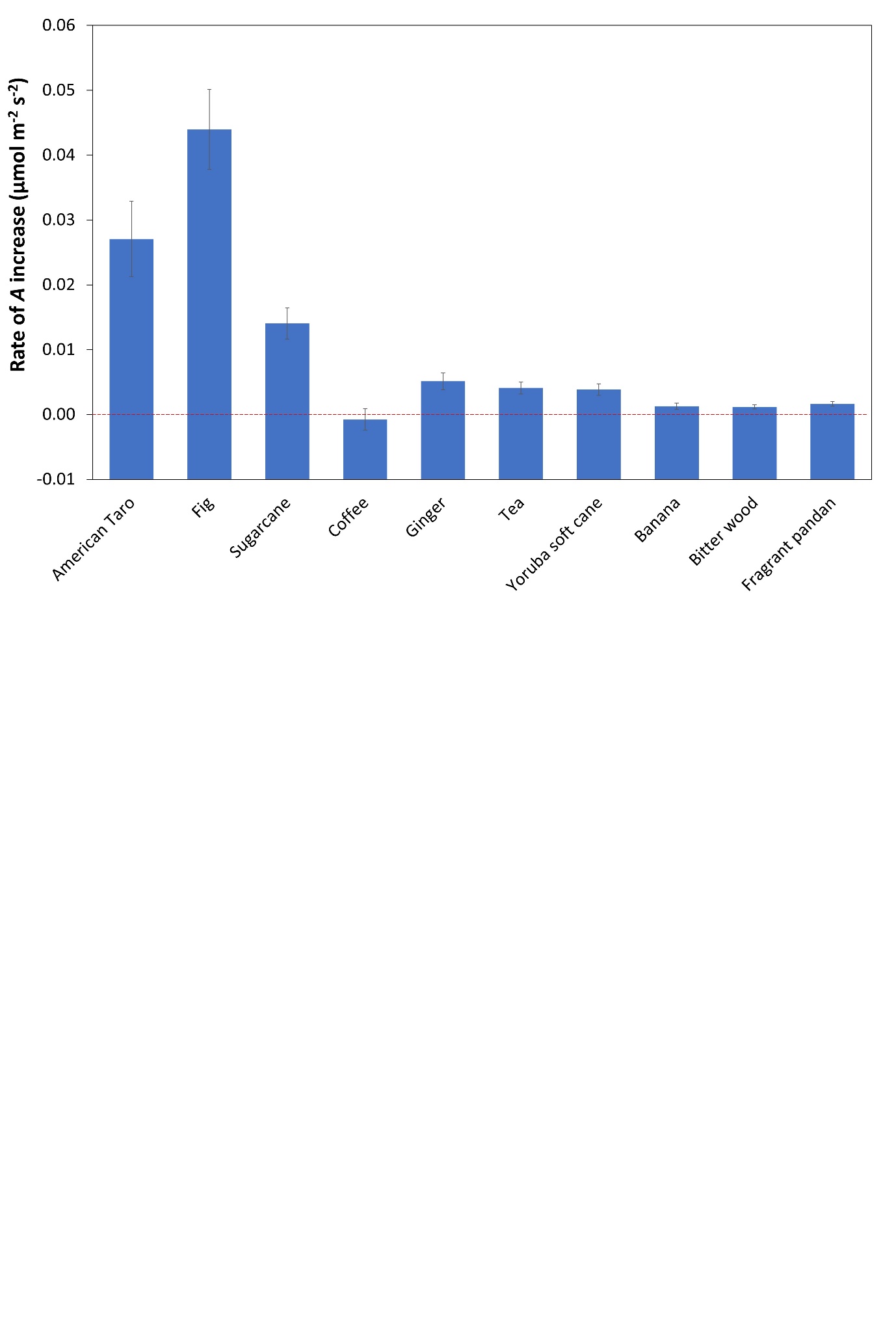


**Figure S3.** Rate of increase in net photosynthesis rate (*A*) during the first 100 seconds of photosynthetic induction in ten tropical species (for details, see Fig. 1). Bars show average values of all measurements conducted per species (data combined for morning, noon, and afternoon), error bars show ± SE (n = 9-15).


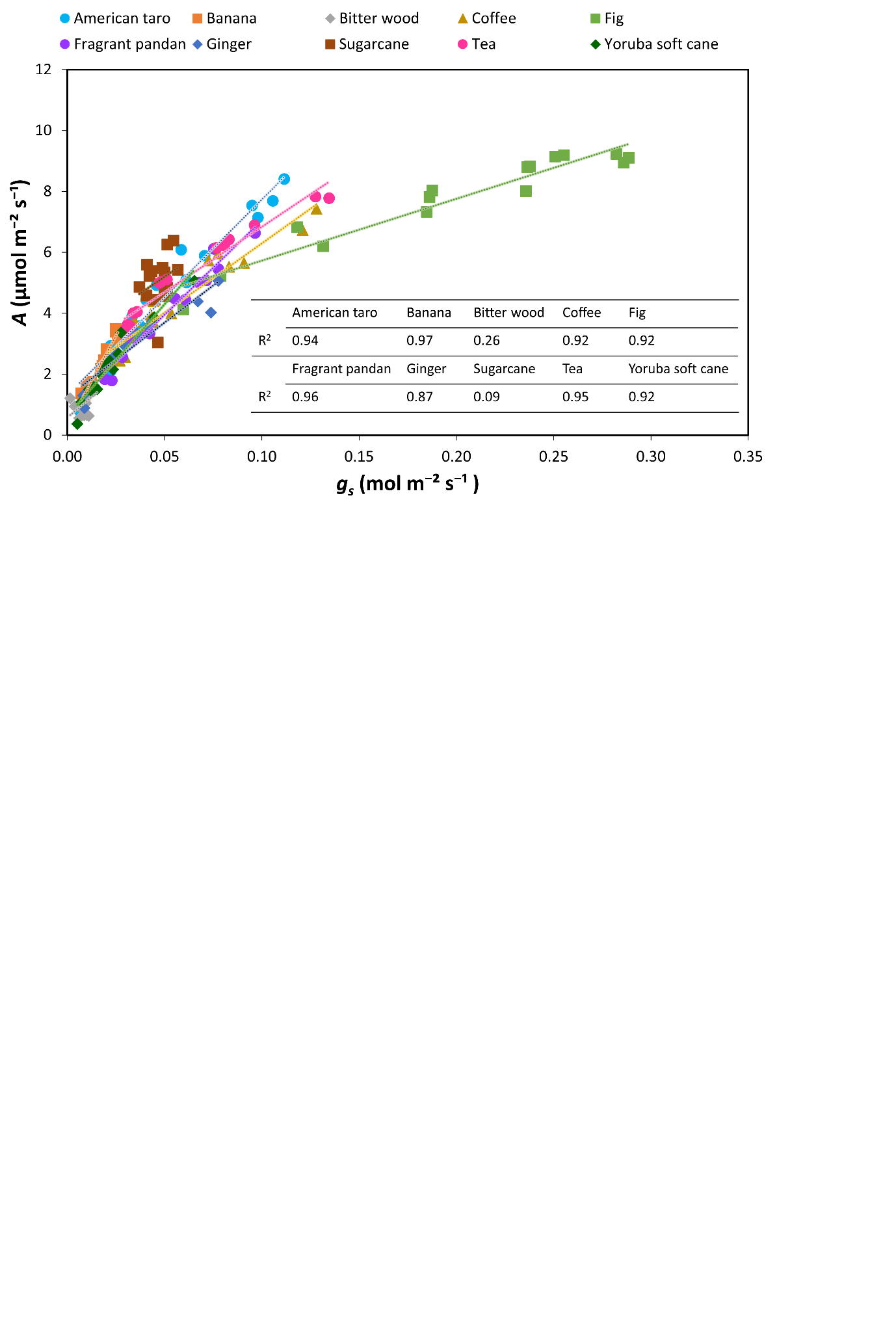


**Figure S4.** Relationship between net photosynthesis rate (*A*) and stomatal conductance (*g_s_*), as measured repeatedly throughout the photoperiod in all ten tropical species (for details, see Fig. 2). Symbols show results per replicate and time point, lines show linear correlations per species. The inset table shows the coefficient of determination (R^2^) of the linear correlation between *A* and *g_s_* per species.


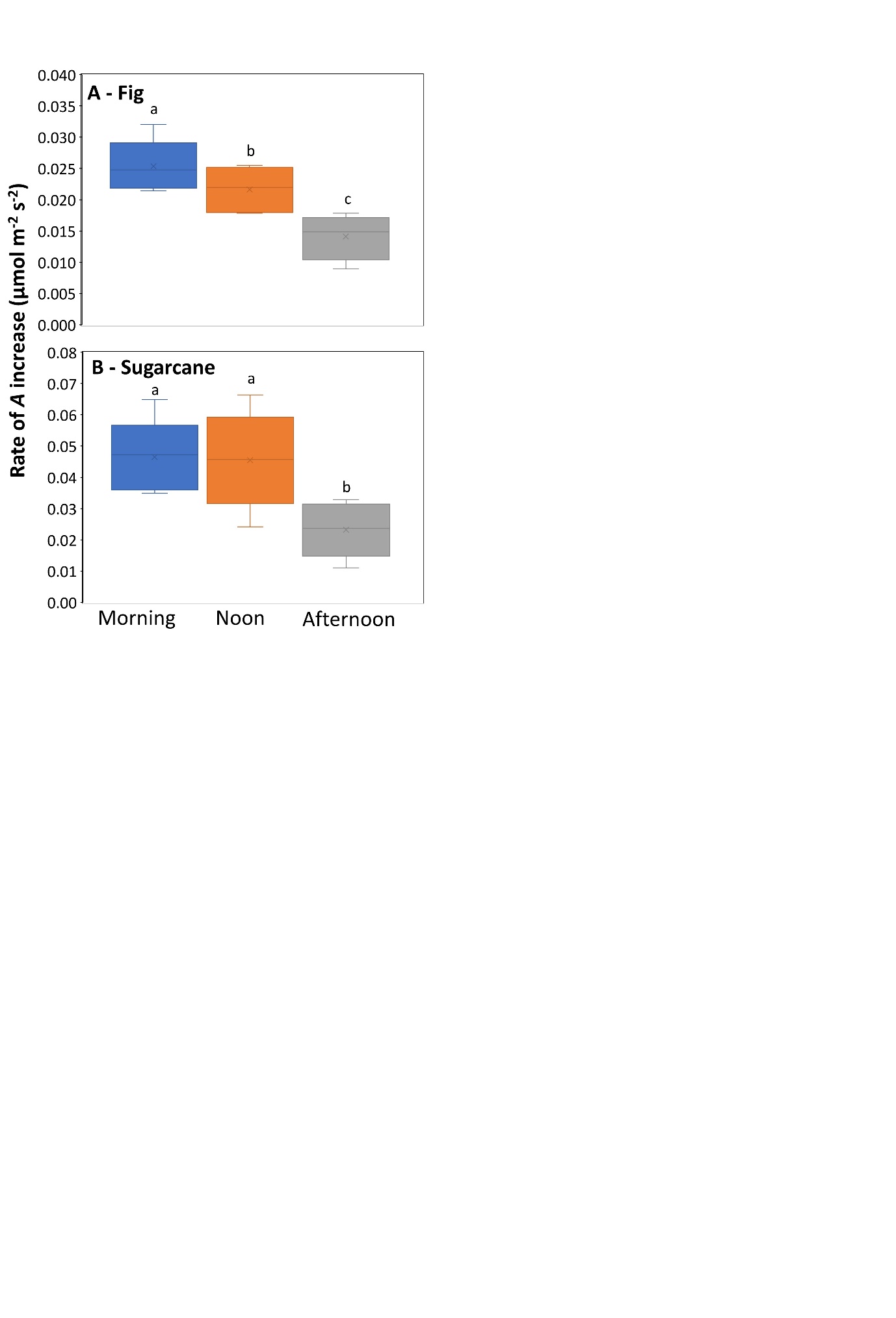


**Figure S5.** Rate of increase in net photosynthesis rate (*A*) during the first 5 minutes of photosynthetic induction at different times of day in fig (A) and sugarcane (B). For details, see Fig. 3. Different letters indicate significant differences (*p*<0.05) between different times of day. The vertical line inside the boxplot represents the median, the x represents the average value, upper and lower limits to the box represent the first and third quantile, respectively, and the whiskers represent the minimum and maximum values.


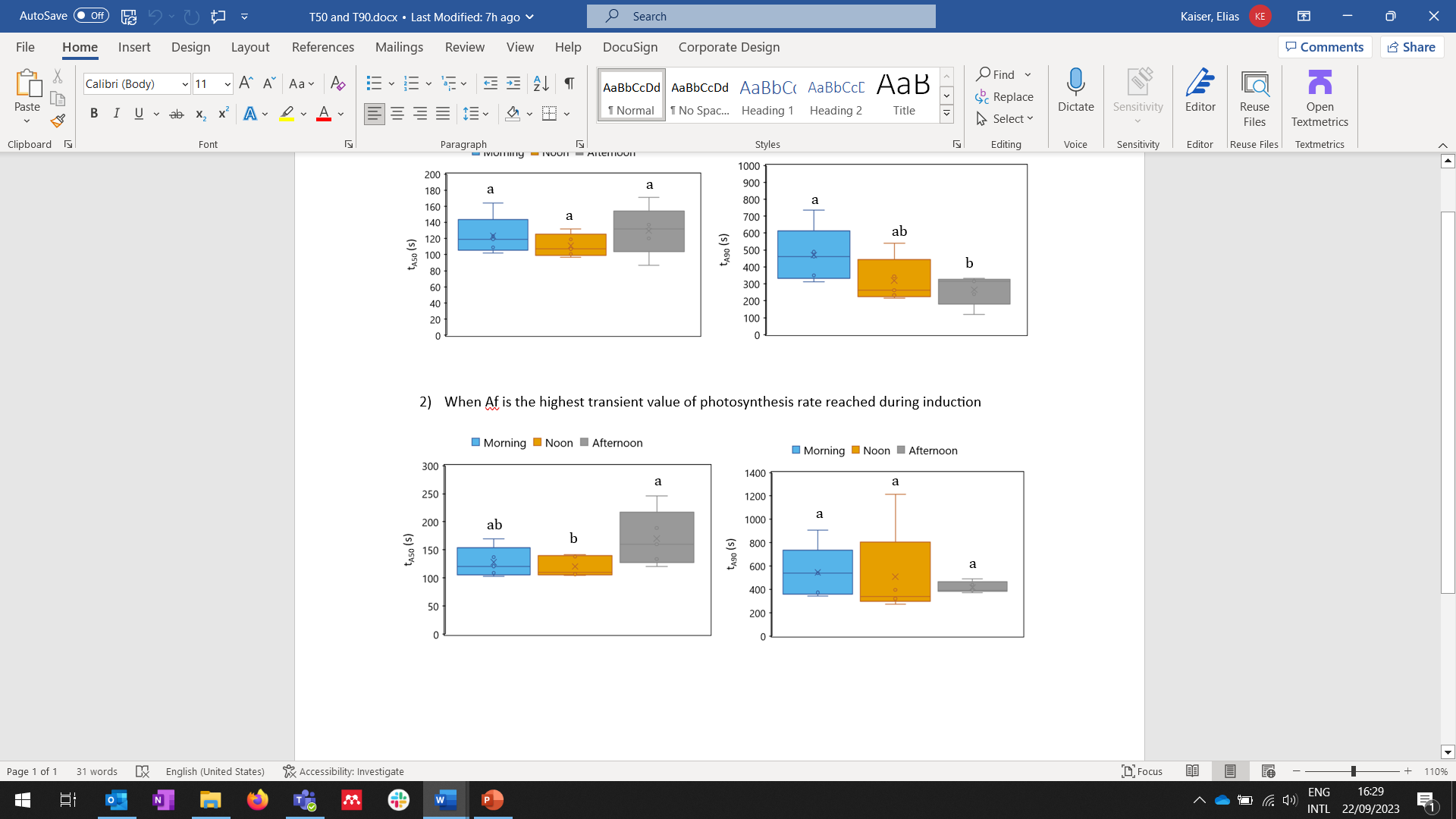


**Figure S6.** Values of t*_A_*_50_ and t*_A_*_90_ in sugarcane (pertaining to Fig. 3B), calculated using the peak value of *A* reached during photosynthetic induction for *A*_f_ (see Eq. 1), instead of the final, steady-state value of *A*.


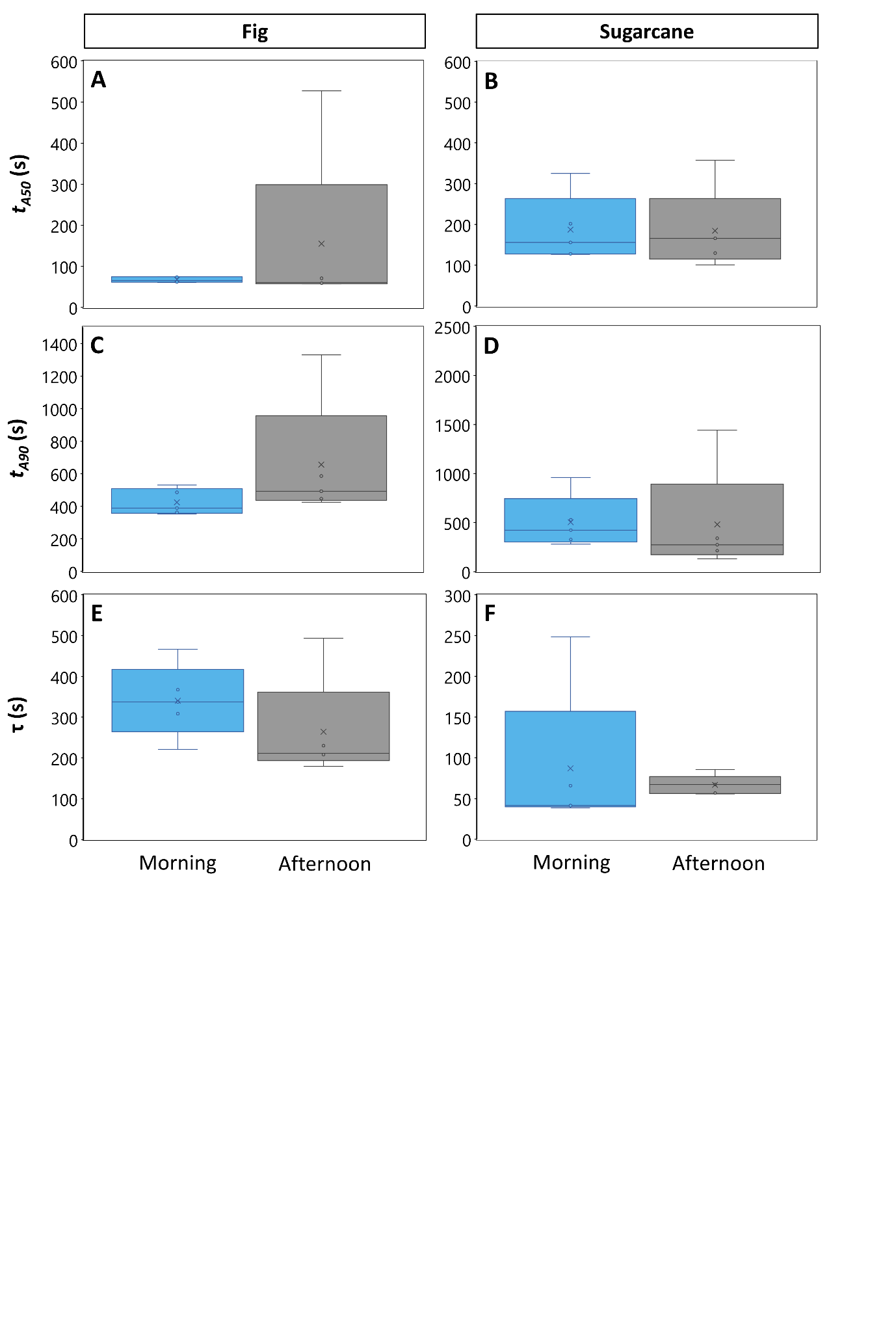


**Figure S7.** Parameters expressing speed of photosynthesis and stomatal conductance response to an irradiance change in fig (C_3_, left panel) and sugarcane (C_4_, right panel); afternoon data as affected by midday dark adaptation (for details, see Fig. 5). A-D, times required to reach 50% (A, B; t_A50_) and 90% (C, D; t_A90_) of final steady-state net photosynthesis rate during photosynthetic induction; E, F, time constant of stomatal opening. The vertical line inside the boxplot represents the median, the x represents the average value, upper and lower limits to the box represent the first and third quantile, respectively, and the whiskers represent the minimum and maximum values. Note that y-axis scales are different between plots.


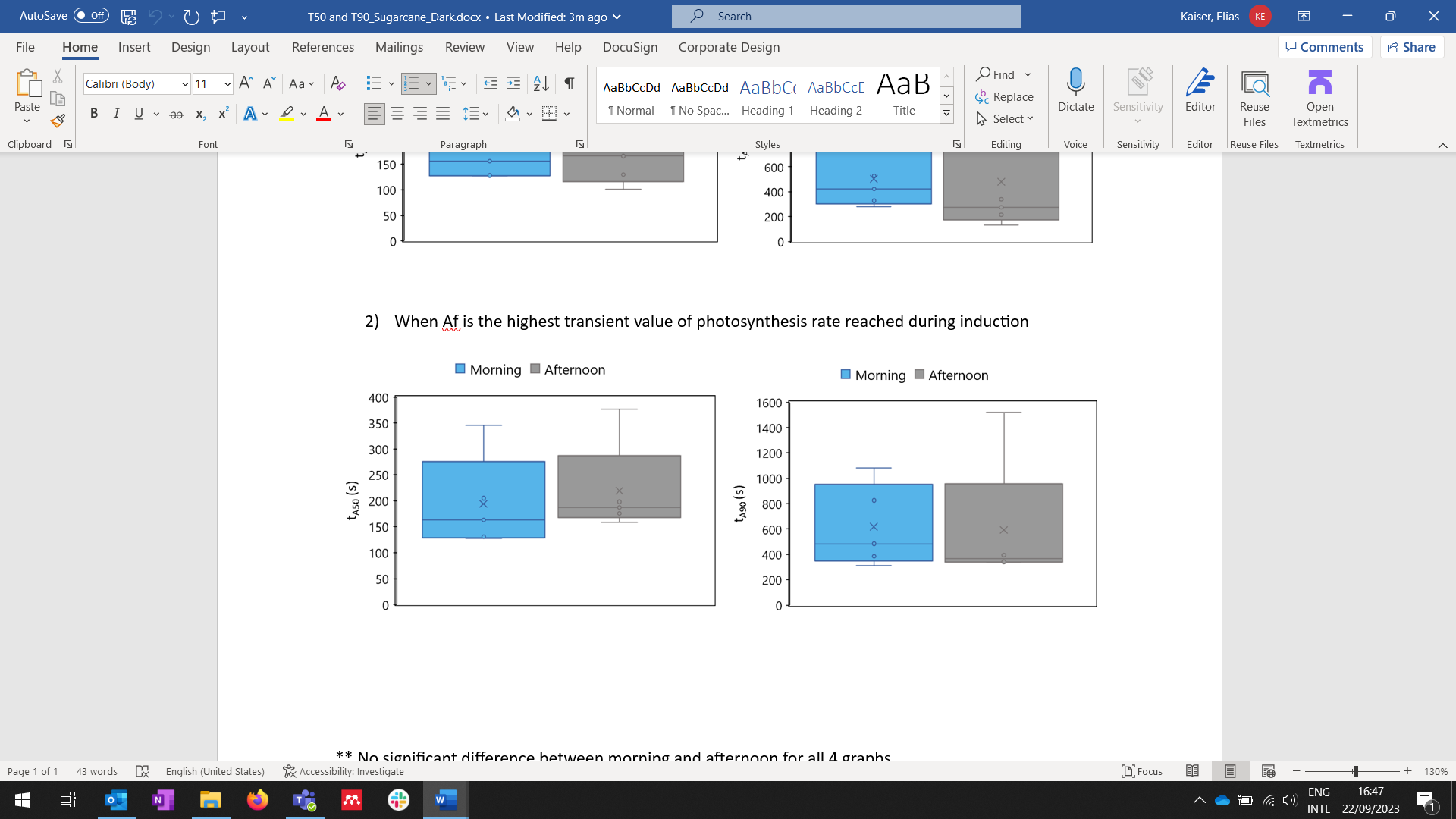


**Figure S8.** Values of t*_A_*_50_ and t*_A_*_90_ in sugarcane (pertaining to Fig. 5B), calculated using the peak value of *A* reached during photosynthetic induction for *A*_f_ (see Eq. 1), instead of the final, steady-state value of *A*.
